# Impact of Psycho-Social Factors on Fatigue among Breast Cancer Patients Who Are Currently Undergoing Radiotherapy

**DOI:** 10.3390/ijerph17176092

**Published:** 2020-08-21

**Authors:** Hyesun Park, Kisook Kim

**Affiliations:** Department of Nursing, Chung-Ang University, Heuksoekro 84, Dongjak gu, Seoul 06974, Korea; april1007@naver.com

**Keywords:** breast cancer, radiotherapy, fatigue, stress, anxiety, depression

## Abstract

Fatigue in breast cancer patients undergoing radiotherapy has been studied less comprehensively than fatigue from chemotherapy. The aim of this study was to test the impact of psycho-social factors on the fatigue among breast cancer patients undergoing radiotherapy. This was a cross-sectional correlational study and participants were 210 breast cancer patients currently undergoing radiotherapy in an outpatient setting in Korea. Data collection was carried out from 22 July to 30 September 2019. The results of this study showed that symptom assessment, anxiety and depression, uncertainty, and perceived stress had a direct effect on the fatigue of breast cancer patients receiving radiotherapy, while social support had an indirect effect. These factors explained 67.2% of the fatigue among the participants. This study confirmed that various interventions for symptom assessment, anxiety and depression, uncertainty, and stress reduction need to be developed to reduce fatigue of breast cancer patients receiving radiotherapy. The present results form the basis for developing such interventions.

## 1. Introduction

Five-year survival rates of breast cancer are steadily increasing globally owing to advances in treatment technology and early screening, with survival rates of 99% in the US and 97% in Korea [1,2].

However, the treatment of breast cancer often combines various methods including surgery, chemotherapy, radiotherapy, targeted therapy, and hormonal therapy. As a result, complications associated with cancer treatment are very diverse and can manifest as long-term physical and mental conditions [3]. Among them, fatigue as a health problem related to the treatment of breast cancer patients was rated at 63.4%, which was higher than the rating of 53.0% for anxiety, depression, and sleep problems [4].

Previous studies on fatigue in breast cancer patients have been primarily conducted with chemotherapy patients [5,6] or after the completion of primary treatment [4,7]. During the cycle of radiotherapy, the fatigue of breast cancer patients continues to increase, with physical symptoms and discomfort also increasing [8,9]. Although fatigue was studied as a factor influencing the quality of life, only a few studies have explored the effects of fatigue on breast cancer patients during the course of radiotherapy [10,11]. Additionally, the research that revealed these factors has been insufficient.

In particular, psychological factors are important variables affecting fatigue in breast cancer patients. Some of these factors include stress due to physical changes [12]; uncertainty, anxiety, and depression [13]; psychological discomfort [14,15]; social support [16]; and family support [17]. Nevertheless, fatigue in breast cancer patients undergoing radiotherapy has been studied less comprehensively than fatigue from chemotherapy. Therefore, in order to reduce the fatigue of breast cancer patients undergoing radiotherapy after chemotherapy, it is important to identify the comprehensive influence of psychological and social factors on these patients, consider their time in treatment, and develop an intervention plan according to their characteristics. 

### Background

The impact of cancer-related fatigue varies in degree from affecting patients’ daily social life to becoming a threat to their quality of life [18]. Fatigue in breast cancer patients is a symptom that is not resolved with the end of the treatment [19], and it affects patients’ adaptation and recovery [20]. It is, however, frequently overlooked because of insufficient understanding and the underestimation of its impact; therefore, fatigue needs to be considered an important problem during the course of breast cancer treatment [21].

While radiotherapy reduces cancer recurrence, it not only affects cancer cells but also destroys normal cells. The procedure of radiotherapy is simpler and shorter than in case of other therapies, but patients still suffer from skin changes, dermatitis, and fatigue during repeated treatment sessions [22,23]. In particular, fatigue is the most frequent symptom of breast cancer patients receiving radiotherapy, and is affected by biological, psychological and social factors [9].

**Figure 1:**
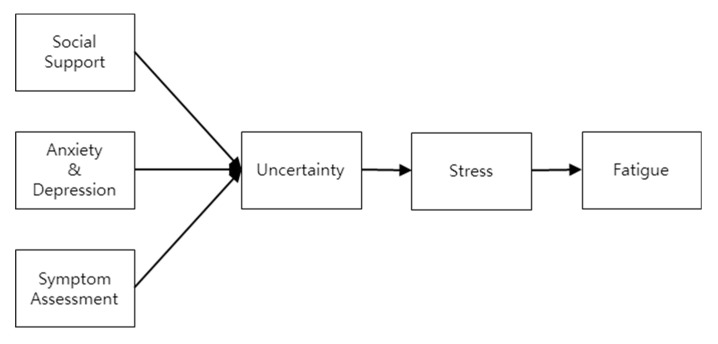
Conceptual framework.

This study was based on stress theory and expanded on previous studies to build a model of factors affecting fatigue in breast cancer patients receiving radiotherapy. In particular, the Generalized Unsafety Theory of Stress (GUTS) stipulates that social and environmental factors, as well as uncertainties and stressors can cause health risks and illness [24]. This theory considers psychological factors and explains the causal relationship between stress and illness more comprehensively than the conventional stress theory [24]. Path setting of the main concept was based on a literature review, and the included factors affecting the fatigue of breast cancer patients undergoing radiotherapy were symptom assessment, anxiety and depression, social support, uncertainty, and stress [20,25,26,27]. Using stress theory and previous studies as its basis, a conceptual framework, presented in Figure 1, was applied in the present study.

## 2. The Study

### 2.1. Aims

To test the impact of psycho-social factors on the fatigue among breast cancer patients who undergo radiotherapy.

### 2.2. Design

This was a cross-sectional correlational study. 

### 2.3. Participants

The participants of this study were breast cancer patients who were receiving radiotherapy at the time of the study. The participants who both understood the purpose of this study and agreed to participate in it were selected. The inclusion criteria of this study were the following:

(1) Being a breast cancer survivor 18 years of age or older, diagnosed with breast cancer, and undergoing only radiotherapy and chemotherapy after surgery;
(2) Understanding the questionnaire;
(3) Voluntarily agreeing to participate in the research.

The exclusion criteria of this study were the following:

(1) Currently diagnosed with a mental health disorder by a psychiatrist and not taking anti-psychotic medications;
(2) Affected by any cancer other than breast cancer.

### 2.4. Sample Size

For structural equation modelling, it is recommended to have at least 200 participants to produce a good index for the maximum likelihood method (ML) [28]. Therefore, in this study, a total of 240 participants were recruited using purposive sampling in consideration of the ideal sample size and the dropout rate due to missing items, while satisfying the minimum recommended sample level for the 12 measurement variables.

### 2.5. Measurements

#### 2.5.1. Validity and Reliability

The variables included in this study were based on stress theory and expanded on previous studies [20,25,26,27] to test the impact of psycho-social factors on fatigue among breast cancer patients who were undergoing radiotherapy. All of the measurement tools used in this study had a previously well-established validity and reliability, and the original author’s permission was obtained by e-mail before the study started.

#### 2.5.2. Symptom Assessment

The Memorial Symptom Assessment Scale-Short Form (MSAS-SF) [29] was used to measure the degree of distress or bother of the participants related to their symptoms. The Korean translated version [30], the reliability and validity of which has been verified, was used in this study. This instrument measures the degree of distress or bother caused by 32 physical and psychological symptoms. If a symptom is identified, the distress or bother associated with its occurrence is rated on a four-point scale ranging from “not at all” (zero points) to “very much” (four points). In this study, higher symptom assessment scores indicate more severe distress or bother experienced from symptoms. Guide instruction of this tool is “If you had the symptom during the past week, please check yes. If you did have the symptom, please check the box that tell us how much the symptom distressed or bothered you.” In a previous study of breast cancer survivors [31], the reliability (Cronbach’s α) of the instrument was 0.85; in this study, the Cronbach’s α was 0.93.

#### 2.5.3. Anxiety and Depression

In this study, the HADS [32] was used to measure anxiety and depression in breast cancer patients. Apart from anxiety and depression of patients, this instrument also evaluates the change in their emotional state. It consists of a total of 14 questions, seven items measuring anxiety and seven items measuring depression. The severity of symptoms is rated on a four-point scale, ranging from zero to three points, in this study, and a higher HADS score indicates more anxiety and depression. The guide instruction is “Tick the box beside the reply that is closest to how you have been feeling in the past week.” An example for anxiety, I feel tense or “wound up”: 3 points (most of time), 2 points (a lot of the time), 1 point (from time to time, occasionally), 0 points (Not at all). For the Korean version of this scale, one study [33] determined that the reliability (Cronbach’s α) was 0.89 for anxiety and 0.86 for depression. In this study, the Cronbach’s α were 0.82 and 0.83 for anxiety and depression, respectively.

#### 2.5.4. Social Support

The Multidimensional Scale of Perceived Social Support (MSPSS) that was developed by Zimet et al. [34] and translated into Korean [35] was used in the present study to measure social support. The MSPSS scale consists of 12 questions, comprising family support, friend support, and special support (significant others). In this study, significant others included health professionals such as physicians and nurses [36]. Each item is measured on a five-point Likert scale, ranging from one point (“not very”) to five points (“very much”) and higher social support scores reflecting higher levels of social support in this study. The guide instruction is “Indicate how you feel about each statement.” One example of this tool is “I get the emotional help and support I need from my family.” Reliability of this scale noted in a previous study was 0.89 [35]. It was 0.88 in this study.

#### 2.5.5. Uncertainty

In this study, uncertainty was measured using the Mishel Uncertainty Illness Scale (MUIS) developed by Mishel [37] and translated and modified for Korean breast cancer patients by Kim and So [36]. It includes 21 questions measuring three sub-concepts of ambiguity, complexity, and unpredictability. Each question is rated on a five-point scale, and the higher the score, the higher the perceived uncertainty in this study. One example of this tool is “ I do not know what is wrong with me” The reliability of this tool, measured by Cronbach’s α, ranged from 0.70–0.91 at the time of its development, and the Cronbach’s α was 0.61–0.79 in Kim and So’s study [36]. In this study, the Cronbach’s α was 0.90 for ambiguity, 0.70 for complexity, and 0.65 for predictability.

#### 2.5.6. Stress

In this study, the stress awareness measurement tool developed by Cohen, Kessler, and Gordon [38] used 10 items of the Perceived Stress Scale (PSS), which were translated and verified by Lee et al. [39]. This tool measures the stress level experienced during the last month with responses rated on a five-point scale ranging from zero points for “never” to four points for “very often”. Higher total stress scores indicate more severity of perceived stress in this study. One example of this tool is “In the last month, how often have you been upset because of something that happened unexpectedly?” At the time of the development of this tool, reliability (Cronbach’s α) was 0.87, and for the Korean version it was 0.82 [39]. In this study, the Cronbach’s α was 0.90.

#### 2.5.7. Fatigue

Fatigue was measured using the Korean version of the FACIT-fatigue scale, a measurement tool developed by the Functional Assessment of Chronic Illness Therapy (FACIT.org). It consists of 13 items, with a total score ranging from 0 to 52. Individual answers are rated on a five-point scale ranging from zero (“not at all”) to four (“very much”), with higher fatigue scores indicating higher fatigue in this study. Guide instruction is “Please circle or mark one number per line to indicate your response as it applies to the past 7 days”, and one example of the tool is “I feel week overall.” At the time of the development of this tool, the reliability indicated by Cronbach’s α was 0.95. In this study, the reliability indicated by the Cronbach’s α was 0.93.

### 2.6. Data Collection

Data collection was carried out in two ways: through web-based and written (paper) surveys conducted from 22 July to 30 September 2019. The web-based survey was conducted by posting information about the research on the largest internet self-help café website in Korea (Breast cancer story) and the survey was made accessible via a hyperlink (Google survey). The written (paper) survey was carried out in nine long-term care hospitals in Seoul and the surrounding area with the permission of each institution, asking the heads of nursing departments to cooperate with the research. The researcher attended a meeting with the survivors and explained the purpose of the research. Written consent was obtained from participants who voluntarily agreed to participate in the study, and they completed a self-report questionnaire. The time required for the completion of the questionnaire was 30–40 min.

There were 75 people who responded by voluntarily accessing the link, and there were no dropouts, so the response rate is 100%. However, four of the web-based questionnaires did not meet the inclusion criteria, so only 71 were used in this study. Cases of duplicate participation were excluded by checking participants’ telephone numbers in the web-based survey. The paper-based questionnaire also recruited people who voluntarily agreed to participate and provided a questionnaire, so there were no dropouts. A total of 165 participants answered the written (paper) survey, after excluding 26 paper-based questionnaires that had more than 10% of non-responses, and 139 questionnaires were used in this study. The final data from 210 participants were included in the analysis.

### 2.7. Ethical Cconsideration

The use of all the research tools in this study was approved by the original author and translator, and the data were collected according to the approval after review by the Institutional Review Board (IRB) of the institution with which the author is affiliated.

### 2.8. Statistical Analysis

The surveyed data were analyzed using SPSS and AMOS 25.0 (IBM Corp., Armonk, NY, USA). Descriptive statistics were used for the general characteristics and disease-related characteristics of the participants. Normality of the sample was verified using mean, standard deviation, skewness, and kurtosis. The structural model verification used the Maximum Likelihood Method, which assumes multivariate normality. The standardized regression weights (S.R.E.) and critical reliability (C. R.) were assessed to examine the significance of the estimated coefficients. To confirm the fit of the model, the chi-square/degrees of freedom ratio (*χ*^2^/*df*), goodness-of-fit (GFI), comparative fit index (CFI), normed fit index (NFI), Tucker–Lewis index (TLI), Root Mean Residual (RMR), and root–mean–square error of approximation (RMSEA) were determined. The direct, indirect, and total effects of the paths in the research model were decomposed and a bootstrapping technique was used to verify the significance of the mediation effect. If the value of *χ*^2^ was non-significant (*p* > 0.05) or *χ*^2^/df value was less than 5, GFI, CFI, NFI, and TLI were 0.90 or greater, and RMSEA was between 0.05 and 0.10, then the model was regarded as appropriate [40].

## 3. Results

### 3.1. Demographic and Cancer-Related Participant Characteristics

General characteristics and disease-related characteristics of this study’s participants are shown in Table 1. The mean age was 48.09 years (±8.50), and most of the participants were university graduates (65.2%). Among the participants, 77.6% had undergone a partial mastectomy. The percentage of participants with either stage 1 or stage 2 breast cancer was 88.9%. The most common period of radiotherapy (34.7%) was three weeks.

### 3.2. Descriptive Analysis of Included Variables

The descriptive statistical results of the variables included in this study are shown in Table 2. In this study, the absolute value of the skewness for all variables did not exceed 2 (−0.82 to 1.64) and the absolute value of the kurtosis did not exceed 7 (−1.05 to 3.26). Therefore, it is confirmed that the maximum likelihood method can be used to apply the structural equation model. 

### 3.3. Structural Model

**Figure 2:**
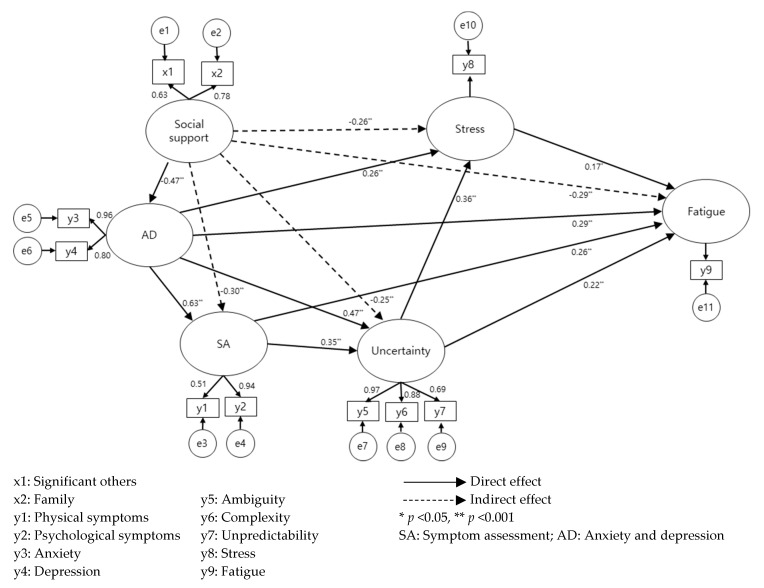
The effect of path diagram of the structural model.

As a result of a confirmatory factor analysis conducted in this study, the results of the goodness-of-fit test for the hypothetical model, and the significance of the estimated coefficients for the analysis of the hypothetical model is shown in Table 3 and Figure 2. The fit index results of the hypothetical model in this study were *χ*^2^ = 99.81 (*p* < 0.001), *χ*^2^/df = 3.22, GFI = 0.92, CFI = 0.95, NFI = 0.93, TLI = 0.91, and RMSEA = 0.10. Since the goodness-of-fit indices meet the recommended criteria, the hypothetical model was confirmed as a structural model without modifying it.

Anxiety and depression had a direct effect (β = 0.63, *p* = 0.004) on symptom assessment, and social support had an indirect effect on symptom assessment (β = −0.30, *p* = 0.004), which was mediated by anxiety and depression. Symptom assessment had a direct effect on uncertainty (β = 0.35, *p* = 0.004), and anxiety and depression had a direct effect (β = 0.47, *p* = 0.004) and an indirect effect (β = 0.22, *p* = 0.004). Social support showed indirect effects on uncertainty (β = −0.25, *p* = 0.006) mediated by symptom assessment and anxiety and depression.

Symptom assessment had a direct effect on stress (β = 0.29, *p* = 0.004) and an indirect effect (β = 0.13, *p* = 0.004), and anxiety and depression had a direct effect (β = 0.26, *p* = 0.007) and an indirect effect (β = 0.43, *p* = 0.004). Social support was found to have indirect effects (β = −0.26, *p* = 0.004) mediated by symptom assessment, anxiety and depression, and uncertainty.

Symptom assessment had a direct effect on fatigue (β = 0.22, *p* = 0.012) and an indirect effect (β = 0.17, *p* = 0.005); anxiety and depression had a direct effect (β = 0.26, *p* = 0.020) and an indirect effect (β = 0.46, *p* = 0.004). Social support showed indirect effects on fatigue (β = −0.29, *p* = 0.007) through symptom assessment, anxiety and depression, uncertainty, and stress. Uncertainty had a direct effect (β = 0.29, *p* = 0.004) and an indirect effect (β = 0.06, *p* = 0.044) on fatigue. Stress had a direct effect on fatigue (β = 0.17, *p* = 0.044). The explanatory power of these variables in relation to fatigue among breast cancer patients undergoing radiotherapy was 67.2%. 

## 4. Discussion

This study applied a hypothetical model and verified its validity in investigating the factors affecting fatigue in breast cancer patients receiving radiotherapy. The results of this study showed that symptom assessment, anxiety and depression, uncertainty, and stress had a direct effect on the fatigue of breast cancer patients receiving radiotherapy while social support had an indirect effect. These factors explained 67.2% of the fatigue of the participants.

The findings of previous studies are consistent with the present findings, which showed that symptom assessment in breast cancer patients affects fatigue (Andic et al., 2019). Symptoms of breast cancer patients receiving radiotherapy include problems with the musculoskeletal, nervous, and reproductive systems. Psychological health problems, such as anxiety and depression, also become more difficult [4]. These symptoms vary throughout the course of treatment and persist even after the end of treatment. The process of treatment needs to include education and management related to symptom assessment of physical changes and management of fatigue.

Previous studies of breast cancer patients showed that the higher the social support, the lower the degree of fatigue [16]. Social support for breast cancer patients receiving hospital treatment was found to show an indirect effect on fatigue [41], and in another study social support, physical symptoms, mood, and family support explained 69.7% of female cancer patients’ fatigue [42]. In addition, fear, sadness, and worry about recurrence have been shown to directly affect the fatigue of breast cancer patients [43]. This may provide a basis for including psychological factors in the interventions for breast cancer patients receiving radiotherapy.

In the present study, symptom assessment and anxiety and depression directly affected uncertainty, while social support had an indirect effect on uncertainty. Uncertainty was a variable directly affecting fatigue. These results are consistent with the conceptual framework suggesting that uncertainty is a key concept in GUTS theory and leads to a stress response causing health problems [24]. Uncertainty was highly correlated with higher levels of fatigue in studies of young breast cancer survivors two to four years after treatment [44]. Uncertainty in breast cancer patients is also related to their quality of life and coping strategies [13]. Anxiety, depression, uncertainty, and social support are important factors for mental health that affect stress and cause health problems [45]. Psychological stress in breast cancer patients after primary care is a factor that increases fatigue [7]. Since the factors affecting fatigue seem to be a complex combination of physical and psychological effects, it is necessary to understand the degree of stress in the early stages of treatment experienced by patients with breast cancer.

In this study, the fatigue of breast cancer patients undergoing radiotherapy was found to be an important health problem that cannot be overlooked. Furthermore, this study confirmed the importance of developing nursing interventions focused on fatigue reduction, considering the impact of many variables, including the impact of psychological factors. Since Korea has a higher incidence of young breast cancer patients in their 40s compared to Western countries due to rapid industrialization [2], it is important to consider that long-term survivors may experience changes in multiple areas of their lives and be exposed to psycho-social sequelae. Therefore, it is necessary to provide breast cancer patients with information to reduce their fatigue after primary treatment and to assist them in adjusting to life after the treatment process, including informing them about what they can anticipate that they will experience as a survivor after treatment. In addition, breast cancer patients under 50 years old tend to experience higher levels of fatigue and pain than older patients [46], while younger breast cancer patients have higher expectations of having a normal life when they are diagnosed than older patients. Considering this, it is necessary to develop and apply different nursing intervention methods that can reduce individual fatigue in the process of receiving radiotherapy.

Consistent with these issues, several interventional studies were conducted to reduce the fatigue of breast cancer patients after primary treatment and during radiotherapy, in which exercise therapy reduced short-term cancer-related fatigue [18,47]. Physical exercise was effective in reducing physical fatigue but not cognitive fatigue [48]. Therefore, taking psychological and physical factors into consideration, breast cancer patients undergoing radiotherapy require more fatigue-related interventions.

### 4.1. Clinical Implications

These findings may contribute to understanding the relationship between variables affecting fatigue and stress in breast cancer patients receiving radiotherapy. By developing and implementing specific nursing intervention programs at each stage of the treatment process, fatigue among breast cancer patients will be reduced, and their quality of life will be improved.

The strength of this study was providing a framework to explain the fatigue of breast cancer patients undergoing radiotherapy and to verify the relationship between the factors associated with it. This study laid the foundation for the knowledge that can contribute to the development of effective interventions to address the fatigue of breast cancer patients. In addition, it is anticipated that the results of this study will contribute to confirming the necessity of developing nursing intervention plans to address the uncertainty and stress of breast cancer patients in order to reduce their fatigue. In addition, guidelines for assessing and predicting the degree of fatigue of these patients will need to be prepared for use in clinical practice. To date, the effectiveness of interventions aiming to reduce fatigue in cancer patients reported by previous research has been inconsistent, and related studies are not sufficient. Therefore, intervention studies based on the patient-specific psycho-social factors investigated in the present study are needed to reduce the fatigue of breast cancer survivors.

### 4.2. Study Limitations

This study has some limitations. Data from the participants were collected in two ways: as a web-based survey and as a written (paper) survey. Therefore, the differences between the characteristics and environmental factors of the two population groups and the convenience of the responses between the groups should be considered. In addition, a purposive selected sample was used in this study, and therefore, it is necessary to be careful when generalizing the results of this study to fatigue in all breast cancer patients receiving radiotherapy. Symptom assessment also includes both physical and psychological symptoms, some of which resemble anxiety and depression. In addition, personal characteristics, demographic factors, and environmental characteristics are expected to have a clear effect on the fatigue of breast cancer patients undergoing radiotherapy, but they have not been included in this analysis. In order to develop comprehensive and generalizable conclusions regarding the sources and nature of fatigue of breast cancer patients receiving radiotherapy, it is necessary to consider symptoms assessment as well as multiple psychosocial factors outlined in this study.

## 5. Conclusions

The purpose of this study was to investigate the causal relationship between psychological factors affecting the fatigue of breast cancer patients receiving radiotherapy, explain the fatigue of breast cancer patients receiving radiotherapy, and verify the theoretical model. The results of this study confirmed that various interventions for symptom assessment of anxiety and depression, uncertainty, and stress reduction need to be developed to reduce fatigue of breast cancer patients receiving radiotherapy. Therefore, this study may contribute to an understanding of the relationship between variables affecting fatigue and stress in breast cancer patients receiving radiotherapy, and to the development and application of a specific nursing intervention programs at each stage of the treatment process that will reduce fatigue and improve the quality of life for breast cancer patients. In addition, comprehensive follow-up studies of various levels of physical health and psychological, social, and environmental factors will be needed in order to comprehensively explain the fatigue of breast cancer patients receiving radiotherapy.

## Figures and Tables

**Table 1:** Demographic and cancer related participant characteristics.

Variables	Classification	n(%) Mean ± SD
Age		48.09 ± 8.50
Spouse	Yes	178 (84.8)
	No	32 (15.2)
Education Level	<=middle school	7 (3.3)
	High school	37 (17.6)
	University	137 (65.2)
	>=Graduate school	29 (13.8)
Job	Have	103 (49.0)
	None	107 (51.0)
Cancer Stage	0	2 (1.0)
	1	104 (49.5)
	2	82 (39.4)
	3	18 (8.7)
	4	2 (1.0)
Type of Mastectomy	Total	47 (22.4)
	Partial	163 (77.6)
Radiation therapy (week)	1st	16 (8.2)
	2nd	46 (23.5)
	3rd	68 (34.7)
	4th	48 (24.5)
	over 5th	18 (9.2)

(N = 210).

**Table 2:** Descriptive analysis of included variables.

Variables		Mean ± SD	Range	Skewness	Kurtosis
SA	Physical symptoms	1.01 ± 0.67	0–4	1.64	3.26
	Psychological symptoms	1.84 ± 1.10	0–4	0.00	−0.98
HADS	Anxiety	1.24 ± 0.58	0–3	0.20	−0.27
	Depression	1.54 ± 0.64	0–3	−0.07	−0.83
Social support	Family	3.98 ± 0.81	1–5	−0.817	0.72
	Friends	2.89 ± 1.08	1–5	0.07	−0.70
	Significant others	2.69 ± 1.21	1–5	0.21	−1.05
Uncertainty	Ambiguity	3.28 ± 0.78	1–5	−0.33	−0.36
	Complexity	3.08 ± 0.67	1–5	−0.27	−0.01
	Unpredictability	3.09 ± 0.68	1–5	−0.36	−0.21
Stress		2.30 ± 0.78	0–4	−0.08	−0.28
Fatigue		2.47 ± 0.94	0–4	−0.54	−0.51

SA: Symptom assessment; HADS: Hospital anxiety and depression scale.

**Table 3:** The suitability and direct, indirect, and total effects of the structural model.

Endogenous Variables	Predictor Variables	SEW (ß)	SE	CR (*p*)	Direct Effect (*p*)	Indirect Effect (*p*)	Total Effect (*p*)	SMC
SA	AD	0.63	0.10	4.77 (<0.001)	0.63 (0.004)	-	0.63 (0.004)	0.315
	Social support	0.21	0.04	2.03 (0.053)	0.21 (0.217)	−0.30 (0.004)	−0.08 (0.615)	
AD	Social support	−0.47	0.05	−4.32 (<0.001)	−0.47 (0.004)	-	−0.47 (0.004)	0.220
Uncertainty	SA	0.35	0.18	4.22 (<0.001)	0.35 (0.004)	-	0.35 (0.004)	0.567
	AD	0.47	0.15	5.17 (<0.001)	0.47 (0.004)	0.22 (0.004)	0.69 (0.004)	
	Social support	−0.08	0.06	−1.04 (0.301)	−0.08 (0.576)	−0.25 (0.006)	−0.33 (0.005)	
Stress	SA	0.29	0.17	3.74 (<0.001)	0.29 (0.004)	0.13 (0.004)	0.42 (.004)	0.646
	AD	0.26	0.13	3.24 (0.001)	0.26 (0.007)	0.43 (0.004)	0.69 (0.004)	
	Social support	−0.06	0.06	−0.85 (0.398)	−0.06 (0.532)	−0.26 (0.004)	−0.32 (0.016)	
	Uncertainty	0.36	0.08	4.69 (<0.001)	0.36 (0.004)	-	0.36 (0.004)	
Fatigue	SA	0.22	0.20	2.91 (0.004)	0.22 (0.012)	0.17 (0.005)	0.39 (0.004)	0.672
	AD	0.26	0.15	3.46 (<0.001)	0.26 (0.020)	0.46 (0.004)	0.72 (0.004)	
	Social support	−0.03	0.06	−0.41 (0.680)	−0.03 (0.835)	−0.029 (0.007)	−0.32 (0.004)	
	Uncertainty	0.29	0.09	3.95 (<0.001)	0.29 (0.004)	0.06 (0.044)	0.36 (0.004)	
	Stress	0.17	0.09	2.29 (0.022)	0.17 (0.044)	-	0.17 (0.044)	

SA: Symptom assessment; AD: Anxiety and depression, SEW: standardized regression weight, CR: critical ratio, SE: standard error, SMC: square multiple correlation, ß: Standardized coefficients.

## References

[1] American Cancer Society (2020). Survival Rates for Breast Cancer. https://www.cancer.org/cancer/breast-cancer/understanding-a-breast-cancer-diagnosis/breast-cancer-survival-rates.html.

[2] National Cancer Information Center (2019). Statistics of Cancer. https://www.cancer.go.kr/lay1/S1T639C641/contents.do.

[3] Lee J.A., Yu J.H., Song Y.M. (2016). Management of Long-Term Breast Cancer Survivors in Korea. J. Korean Med. Assoc..

[4] Ligt K.M.D., Heins M., Verloop J., Smorenburg C.H., Korevaar J.C., Siesling S. (2019). Patient-Reported Health Problems and Health Care Use After Treatment for Early Breast Cancer. Breast.

[5] Whisenant M., Wong B., Mitchell S.A., Beck S.L., Mooney K. (2017). Distinct Trajectories of Fatigue and Sleep Disturbance in Women Receiving Chemotherapy for Breast Cancer. Oncol. Nurs. Forum.

[6] Zhang B., Dong J.N., Sun P., Feng C., Liu Y.C. (2017). Effect of Therapeutic Care for Treating Fatigue in Patients with Breast Cancer Receiving Chemotherapy. Medicine.

[7] Ploos van Amstel F.K.P., van den Berg S.W., van Laarhoven H.W., Gielissen M.F., Prins J.B., Ottevanger P.B. (2013). Distress Screening Remains Important During Follow-up After Primary Breast Cancer Treatment. Support. Care Cancer.

[8] Andic F., Miller A.H., Brown G., Chu L., Lin J., Liu T., Sertdemir Y., Torres M.A. (2020). Instruments for Determining Clinically Relevant Fatigue in Breast Cancer Patients During Radiotherapy. Breast Cancer.

[9] Muszalik M., Kołucka-Pluta M., Kędziora-Kornatowska K., Robaczewska J. (2016). Quality of Life of Women with Breast Cancer Undergoing Radiotherapy Using the Functional Assessment of Chronic Illness Therapy-Fatigue Questionnaire. Clin. Interv. Aging.

[10] Abrahams H.J.G., Gielissen M.F.M., Verhagen C.A.H.H.V.M., Knoop H. (2018). The Relationship of Fatigue in Breast Cancer Survivors with Quality of Life and Factors to Address in Psychological Interventions: A Systematic Review. Clin. Psychol. Rev..

[11] Reinertsen K.V., Engebraaten O., Loge J.H., Cvancarova M., Naume B., Wist E., Edvardsen H., Wille E., Bjøro T., Kiserud C.E. (2017). Fatigue During and After Breast Cancer Therapy a Prospective Study. J. Pain Symptom Manag..

[12] Kagee A., Roomaney R., Knoll N. (2018). Psychosocial Predictors of Distress and Depression Among South African Breast Cancer Patients. Psychooncology.

[13] Pahlevan Sharif S.S., Ahadzadeh A.S., Perdamen H.K. (2017). Uncertainty and Quality of Life of Malaysian Women with Breast Cancer: Mediating Role of Coping Styles and Mood States. Appl. Nurs. Res..

[14] Fradelos E.C., Papathanasiou I.V., Veneti A., Daglas A., Christodoulou E., Zyga S., Kourakos M. (2017). Psychological Distress and Resilience in Women Diagnosed with Breast Cancer in Greece. Asian Pac. J. Cancer Prev..

[15] Kishan A.U., Wang P.C., Sharif J., Kupelian P.A., Steinberg M.L., McCloskey S.A. (2016). Clinical Indicators of Psycho- Social Distress Predict for Acute Radiation-Induced Fatigue in Patients Receiving Adjuvant Radiation Therapy for Breast Cancer: An Analysis of Patient-Reported Outcomes. Int. J. Radiat. Oncol. Biol. Phys..

[16] Schmidt M.E., Wiskemann J., Schneeweiss A., Potthoff K., Ulrich C.M., Steindorf K. (2018). Determinants of Physical, Affective, and Cognitive Fatigue During Breast Cancer Therapy and 12 Months Follow-Up. Int. J. Cancer.

[17] Fagundes C.P., Lindgren M.E., Shapiro C.L., Kiecolt-Glaser J.K. (2012). Child Maltreatment and Breast Cancer Survivors: Social Support Makes a Difference for Quality of Life, Fatigue and Cancer Stress. Eur. J. Cancer.

[18] Lipsett A., Barrett S., Haruna F., Mustian K., O’Donovan A. (2017). The Impact of Exercise During Adjuvant Radiotherapy for Breast Cancer on Fatigue and Quality of Life: A Systematic Review and Meta-Analysis. Breast.

[19] Matias M., Baciarello G., Neji M., Di Meglio A., Michiels S., Partridge A.H., Bendiane M.K., Fizazi K., Ducreux M., Andre F. (2019). Fatigue and Physical Activity in Cancer Survivors: A Cross-Sectional Population–Based Study. Cancer Med..

[20] Bower J.E., Wiley J., Petersen L., Irwin M.R., Cole S.W., Ganz P.A. (2018). Fatigue After Breast Cancer Treatment: Biobehavioral Predictors of Fatigue Trajectories. Health Psychol..

[21] Pearson E.J.M., McKinstry C.E., Morris M.E. (2015). Which Clinical Practice Guideline for Cancer-Related Fatigue Is the Most Suitable for Application in Australia?. Asia Pac. J. Clin. Oncol..

[22] Andersen E.R., Eilertsen G., Myklebust A.M., Eriksen S. (2018). Women’s Experience of Acute Skin Toxicity Following Radiation Therapy in Breast Cancer. J. Multidiscip. Healthc..

[23] Radvansky L.J., Pace M.B., Siddiqui A. (2013). Prevention and Management of Radiation-Induced Dermatitis, Mucositis, and Xerostomia. Am. J. Health Syst. Pharm..

[24] Brosschot J.F., Verkuil B., Thayer J.F. (2017). Exposed to Events That Never Happen: Generalized Unsafety, the Default Stress Response, and Prolonged Autonomic Activity. Neurosci. Biobehav. Rev..

[25] Park J.H., Bae S.H., Chun M., Jung Y.S., Jung Y.M. (2015). Factors Influencing Elevated Distress Scores at the End of Primary Treatment of Breast Cancer. Asian Oncol. Nurs..

[26] Oh Y.K., Hwang S.Y. (2018). Impact of Uncertainty on the Quality of Life of Young Breast Cancer Patients: Focusing on Mediating Effect of Marital Intimacy. J. Korean Acad. Nurs..

[27] Shand L.K., Cowlishaw S., Brooker J.E., Burney S., Ricciardelli L.A. (2015). Correlates of Post Traumatic Stress Symptoms and Growth in Cancer Patients: A Systematic Review and Meta-Analysis. Psychooncology.

[28] Weston R., Gore P.A. (2006). A Brief Guide to Structural Equation Modeling. Couns. Psychol..

[29] Chang V.T., Hwang S.S., Feuerman M., Kasimis B.S., Thaler H.T. (2000). The Memorial Symptom Assessment Scale Short Form(MSAS-SF) Validity and Reliability. Cancer.

[30] Nho J.H., Kim S.R., Chang V.T., Nam J.H. (2018). Reliability and Validity of the Korean Memorial Symptom Assessment Scale-Short Form in Gynecological Cancer Patients. J. Pain Symptom Manag..

[31] Thompson P. (2007). The Relationship of Fatigue and Meaning in Life in Breast Cancer Survivors. Oncol. Nurs. Forum.

[32] Zigmond A.S., Snaith R.P. (1983). The Hospital Anxiety and Depression Scale. Acta Psychiatr. Scand..

[33] Oh S.M., Min K.J., Park D. (1999). A Study on the Standardization of the Hospital Anxiety and Depression Scale for Koreans. J. Korean Neuropsychiatr. Assoc..

[34] Zimet G.D., Dahlem N.W., Zimet S.G., Farley G.K. (1988). The Multidimensional Scale of Perceived Social Support. J. Pers. Assess..

[35] Shin J.S., Lee Y.B. (1999). The Effects of Social Supports on Psychosocial Well-Being of the Unemployed. Korean J. Soc. Welf..

[36] Kim H.Y., So H.S. (2012). A Structural Model for Psychosocial Adjustment in Patients with Early Breast Cancer. J. Korean Acad. Nurs..

[37] Mishel M.H. (1981). The Measurement of Uncertainty in Illness. Nurs. Res..

[38] Cohen S., Kessler R.C., Gordon L.U. (1997). Measuring Stress: A Guide for Health and Social Scientists.

[39] Lee J., Shin C., Ko Y.H., Lim J., Joe S.H., Kim S., Jung I.K., Han C. (2012). The Reliability and Validity Studies of the Korean Version of the Perceived Stress Scale. Korean J. Psychosom. Med..

[40] Hur J. (2013). AMOS for Structural Equation Model.

[41] So W.K., Leung D.Y., Ho S.S., Lai E.T., Sit J.W., Chan C.W. (2013). Associations Between Social Support, Prevalent Symptoms and Health-Related Quality of Life in Chinese Women Undergoing Treatment for Breast Cancer: A Cross-Sectional Study Using Structural Equation Modelling. Eur. J. Oncol. Nurs..

[42] Kim K., Lee M., Kwak Y., Kim J. (2014). Factors Affecting the Fatigue of Hospitalized Women Cancer Patients Receiving Chemotherapy. Asian Oncol. Nurs..

[43] Edib Z., Kumarasamy V., Binti Abdullah N., Rizal A.M., Al-Dubai S.A.R. (2016). Most prevalent unmet supportive care needs and quality of life of breast cancer patients in a tertiary hospital in Malaysia. Health Qual. Life Outcomes.

[44] Hall D.L., Mishel M.H., Germino B.B. (2014). Living with Cancer-Related Uncertainty: Associations with Fatigue, Insomnia, and Affect in Younger Breast Cancer Survivors. Support. Care Cancer.

[45] Harvey S.B., Modini M., Joyce S., Milligan-Saville J.S., Tan L., Mykletun A., Bryant R.A., Christensen H., Mitchell P.B. (2017). Can Work Make You Mentally Ill? A Systematic Meta Review of Work Related Risk Factors for Common Mental Health Problems. Occup. Environ. Med..

[46] Levkovich I., Cohen M., Alon S., Kuchuk I., Nissenbaum B., Evron E., Pollack S., Fried G. (2018). Symptom Cluster of Emotional Distress, Fatigue and Cognitive Difficulties Among Young and Older Breast Cancer Survivors: The Mediating Role of Subjective Stress. J. Geriatr. Oncol..

[47] Juvet L.K., Thune I., Elvsaas I.K.Ø., Fors E.A., Lundgren S., Bertheussen G., Leivseth G., Oldervoll L.M. (2017). The Effect of Exercise on Fatigue and Physical Functioning in Breast Cancer Patients During and After Treatment and at 6 Months Follow-up: A Meta-Analysis. Breast.

[48] Van Vulpen J.K., Peeters P.H., Velthuis M.J., van der Wall E., May A.M. (2016). Effects of Physical Exercise During Adjuvant Breast Cancer Treatment on Physical and Psychosocial Dimensions of Cancer-Related Fatigue: A Meta-Analysis. Maturitas.

